# High-performance nanotube-enhanced perovskite photodetectors

**DOI:** 10.1038/srep45543

**Published:** 2017-03-30

**Authors:** Ibrahima Ka, Luis Felipe Gerlein, Riad Nechache, Sylvain G. Cloutier

**Affiliations:** 1 Department of Electrical Engineering, École de Technologie Supérieure, 1100 Notre-Dame Ouest, Montréal, Québec, H3C 1K3, Canada

## Abstract

Organic-inorganic perovskites have already shown a tremendous potential for low-cost light-harvesting devices. Yet, the relatively low carrier mobilities in bulk perovskites still prevent large-area devices with performances competing with state-of-the-art technologies. Here, we tackle this fundamental challenge by incorporating single-wall carbon nanotubes within a perovskite matrix by means of a simple two-step method in ambient air. Using this nano-engineered hybrid film, we demonstrate large-area photodetectors with responsivities up-to 13.8 A.W^−1^ and a broad spectral response from 300 to 800 nm, indicating that photocurrent generation arises from the charge transfer from the perovskite matrix to the embedded nanotube network. As the nanotubes facilitate the carrier extraction, these photodetectors also show a fast response time of 10 ms. This is significantly faster than most of previous reports on perovskite-based photodetectors, including devices with much smaller photosensitive areas. This approach is also well-suited for large-scale production of other perovskite-based light-harvesting devices.

Today, a proven strategy to procure better low cost materials is the combination of different nanomaterials^1^,^2^,^3^,^4^,^5^,^6^,^7^. Such hybrid nano-engineered materials are of great interest because they can often synergistically and controllably associate the advantageous physical, chemical and optical properties from each constituent to achieve better, novel and tunable functionalities^2^,^3^,^4^,^5^,^6^,^7^. In such hybrid systems, carbon nanotubes (CNTs) provide a useful building block based on their high electrical conductivity and unique physical properties^4^,^6^,^7^. Indeed, CNTs associated with quantum dots have previously shown significant improvement in photosensitizing device performances^6^,^7^. While solution processing techniques have succeeded in synthesizing hybrid nano-engineered materials for optoelectronic applications, many key parameters including compatibility, interface engineering, surface treatment and processability are essential to reach the best device performances^8^,^9^,^10^.

More recently, organometallic halide perovskites (e.g., CH_3_NH_3_PbA_3−x_B_x_, A and B = I, Cl, Br) have shown tremendous potential for optoelectronic device integration, including light-emitting diodes, solar cells and photodetectors^11^,^12^,^13^,^14^,^15^. For example, the power-conversion efficiencies from organometallic halide perovskite solar cells have increased from 3.8% in 2009^14^ to 22.1% in 2016^15^. This spectacular progress is largely attributed to improved processability and longer charge carrier lifetimes directly related to increased grain size and the reduced grain boundaries^16^,^17^. These advances have been transposed to perovskite-based photodetectors, where responsivities between 10^2^ and 10^6^ A.W^−1^ have been reported with various photodetector architectures^3^,^4^,^18^,^19^. Very recently, a photoconducting-type device has been investigated using solution-processed graphite-perovskite hybrid as a photoactive material^1^. The dependence of the device performance with the channel length has been demonstrated. A highest responsivity of ~0.8 A.W^−1^ is obtained with the shortest channel length of 40 μm^1^. Such devices operating with micron-scale photosensitive areas are scarcely practical. Therefore, new strategies are required to achieve practical low-cost perovskite-based photodetectors combining high responsivities with fast response times.

Here, we report on introducing nanotubes within a perovskite matrix using a facile and highly-reproducible two-step process to synthesize a hybrid film based on a combination of single-wall carbon nanotubes (SWCNTs) and CH_3_NH_3_PbI_3−x_Cl_x_ (MAPbI_3−x_Cl_x_) perovskite. To demonstrate the enhanced capabilities of these hybrid nano-engineered films, we produced large-area (0.1 cm^2^) and broadband photodetectors operating from 300 nm to 800 nm, with responsivities up-to 13.8 A.W^−1^ and response times as short as 10 ms, which is faster than most previous works on perovskite photodetectors^1^,^2^,^3^,^4^. These spectacular performances stem from the synergetic contributions of the SWCNTs and the perovskite matrix to significantly improve the overall properties of the hybrid material compared to the bulk perovskite films. Our results suggest that the generation of charge carriers under illumination originates in the perovskite, and their extraction occurs by transfer through the SWCNTs. However, we also observe that an excessive concentration of nanotubes can impede the device performance as it prevents a deeper penetration of the spin-coated perovskite precursor atop the sprayed-on nanotube mesh. We firmly believe that combining the outstanding photo-generation capability of perovskites with the superior carrier-extraction achieved with carbon nanotubes will rapidly have a transformative impact on the field of low-cost and large-area light-harvesting devices.

## Results and Discussion

### Synthesis and characterization of the nano-engineered SWCNT/perovskite hybrid materials

**Figure 1:**
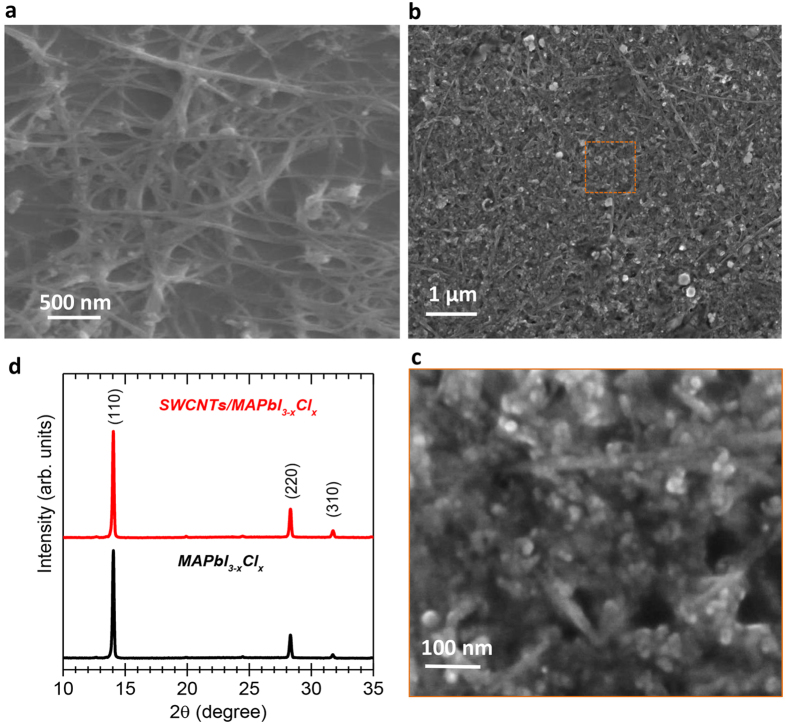
Material characterization. Typical top-view scanning electron microscopy (SEM) images of a bundled mesh of SWCNT deposited by spray coating (**a**) before and (**b**) after the deposition of the nanocrystals of MAPbI_3−x_Cl_x_. (**c**) High-resolution top-view SEM image of the SWCNT/MAPbI_3−x_Cl_x_ hybrid film. (**d**) X-ray diffraction measurements of the nanocrystals of MAPbI_3−x_Cl_x_ (black) with and (red) without the SWCNT.

A typical scanning electron microscopy (SEM) micrograph of the intertwined nanotube mesh deposited on glass using spray-coating is presented in Fig. 1a. These intertwined nanotube networks consist of interconnected bundles of 40 to 100 nm in diameter. Fortunately, the spray-coating process allows precise control of the nanotube density and its electrical resistance. Indeed, Supplementary Figure S1 shows different SWCNT-based film densities identified with their respective electrical-resistance values. As we will demonstrate, it is clear that optimizing the SWCNT film morphology has direct consequences on the performance of the devices.

To prepare the solution precursor for the MAPbI_3−x_Cl_x_ perovskite, we have slightly modified a previously-published one-step approach^20^ (see Methods). After stirring the precursors for 30 min, the solution is spin-coated directly atop the SWCNT mesh and annealed in ambient atmosphere at 120 °C for 15 min to form the MAPbI_3−x_Cl_x_ perovskite matrix. Figure 1(b,c) show the SEM images of the final SWCNT/MAPbI_3−x_Cl_x_ hybrid film at different resolutions. The higher resolution image in Fig. 1c reveals clearly that the MAPbI_3−x_Cl_x_ perovskite consists of nanocrystals smaller than 20 nm closely-connected to the bundles of SWCNTs to form the hybrid system. Given the morphology of the hybrid film, it is difficult to estimate the percentage of the perovskite closely-connected to the SWCNTs. However, it is clear from SEM observation that the porous structure of the SWCNT film is almost completely filled by small perovskites crystals. Most importantly, X-ray diffraction results in Fig. 1d reveal no noticeable differences between the crystalline structures for the perovskite produced with and without the SWCNT mesh. In both cases, the main peaks around 14.0°, 28.3° and 31.7° are consistent with previous reports^20^,^21^, suggesting that the perovskite crystalizes in single phase with a high crystal quality. Moreover, the absence of the PbI_2_ peaks around 11.2^°^ and 12.6° confirms the complete conversion of the precursor^21^. Obviously, we could not exclude a difference in perovskite crystal size and morphology with and without the SWCNTs, as the SWCNT film restricts the growth of the perovskite. Such influence in the grain size is demonstrated in perovskite within mesoporous TiO_2_ scaffold compared with perovskite on planar TiO_2_ layer^22^.

**Figure 2:**
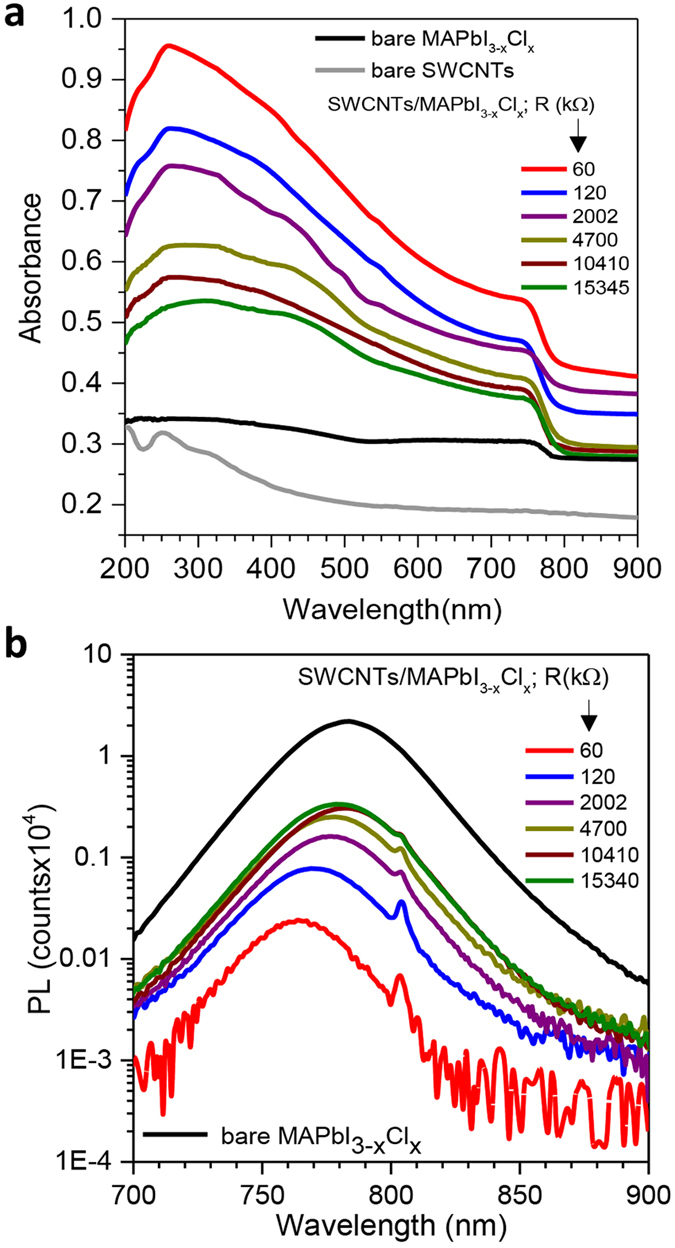
Absorption and photoluminescence measurements. (**a**) UV-vis absorption spectra of different SWCNT/MAPbI_3−x_Cl_x_ hybrid films on quartz using various densities of SWCNT (identified by their electrical resistances). (**b**) Photoluminescence spectra of MAPbI_3−x_Cl_x_ perovskite compared to SWCNT/MAPbI_3−x_Cl_x_ hybrid made with various densities of SWCNT (identified by their electrical resistances).

Optical absorption is a key parameter when developing high-sensitivity photodetectors or light-harvesting devices. To investigate the effect of the embedded SWCNTs on the light absorption properties of the hybrid material, we performed spectrophotometric measurements on different SWCNTs/perovskite films. Figure 2a compares the optical absorption of perovskite films formed atop SWCNT mesh-electrodes with different densities (indicated by their different electrical resistance values), together with SWCNT and MAPbI_3−x_Cl_x_ reference samples. As expected, the hybrid SWCNT/perovskite films absorb more efficiently than both the SWCNT and MAPbI_3−x_Cl_x_ reference samples and their absorbance depends on the density of SWCNTs. Having synthesized samples using SWCNT films with electrical resistances varying over three orders of magnitude, the absorbance can be seen to increase monotonically with the decrease of the resistance of the SWCNT film due to an increase of both density and overall film thickness. This enhanced light absorption properties, which is essential for photonic device applications, can be explained by the high diffusivity of the light promoted by the surface roughening with introducing SWCNTs. We also observe that the absorption edge of the hybrid films spectra (around 790 nm or 1.57 eV) matches the bare perovskite, which confirms that the perovskite nanocrystals clearly dominate the photogeneration process in the hybrid films.

Meanwhile, photoluminescence (PL) spectroscopy results depicted in Fig. 2b provide critical information on the charge carrier extraction properties in the hybrid films. Given the high luminescence of MAPbI_3−x_Cl_x_, the quenching of its photoluminescence intensity can probe the charge-extraction ability of the hybrid films. As expected, the bare MAPbI_3−x_Cl_x_ shows an intense emission centered at 782 nm, which is consistent with the absorption edge of bare MAPbI_3−x_Cl_x_ (cf. Fig. 2a). In contrast, the PL from the MAPbI_3−x_Cl_x_ significantly decreases when the perovskite is mixed with the SWCNT. Indeed, the emission intensity can decrease by up-to 3 orders of magnitude atop SWCNT compared with bare perovskite. It is important to note that higher densities of the SWCNT bundles (i.e. lower resistance) quench the PL emission due to better charge transfer from the perovskite to the SWCNT. While no literature can be found on using SWCNTs within a perovskite matrix, this observation is consistent with results previously reported on perovskite photovoltaic devices using nanostructured carbon-based electrodes^1^,^23^. The pronounced PL quenching observed in our SWNT/perovskite films highlights the role of the carbon nanotube network as charge-carrier separator and extractor^1^,^23^. This enhanced charge collection in the hybrid films can be directly attributed to the increased interaction between the small perovskite nanocrystals and the embedded SWCNT network.

### SWCNT-enhanced MAPbI_3−x_Cl_x_ perovskite photodetectors

**Figure 3:**
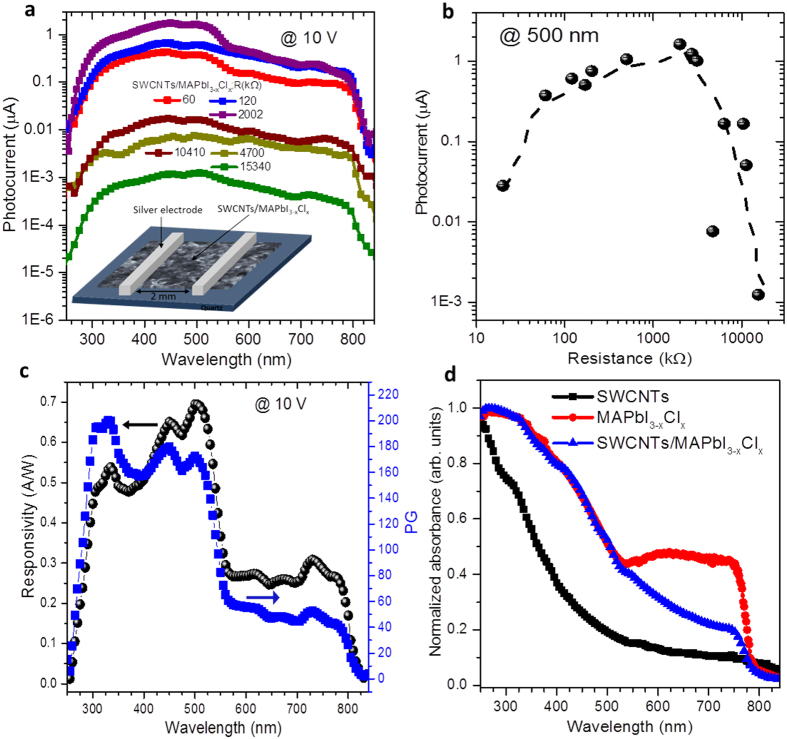
SWCNT/MAPbI_3−x_Cl_x_ perovskite hybrid photodetector. (**a**) Photocurrent spectra of different SWCNT/MAPbI_3−x_Cl_x_ hybrid devices made with various densities of SWCNT (identified by their electrical resistances). (**b**) Photocurrent at 500 nm of the different devices as a function of the electrical resistance of the spray-coated SWCNT films. (**c**) Responsivity and photoconductive gain spectra of our best device. (**d**) Comparison between normalized absorbance of the SWCNT mesh, MAPbI_3−x_Cl_x_ and SWCNT/MAPbI_3−x_Cl_x_ hybrid.

**Figure 4:**
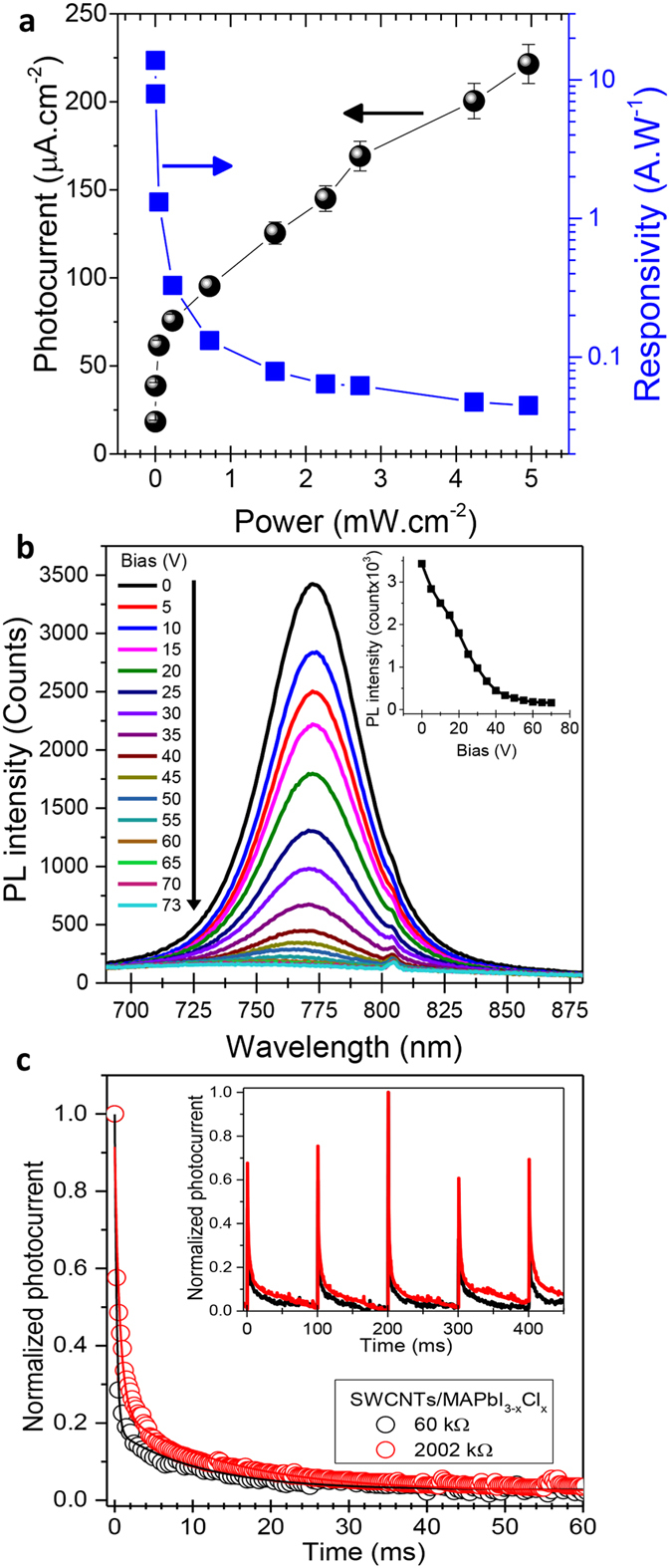
Photocurrent under illumination, photoluminescence quenching with applied bias and photocurrent decay. (**a**) Photocurrent and the corresponding responsivity of the SWCNT/MAPbI_3−x_Cl_x_ hybrid photodetector as a function of the illumination power of a 532 nm laser. (**b**) Photoluminescence of the SWCNT/MAPbI_3−x_Cl_x_ hybrid photodetector with applied bias ranging from 0 to 73 V. (**c**) Photocurrent decay of the SWCNT/MAPbI_3−x_Cl_x_ hybrid photodetector under illumination with a 532 nm nanosecond pulsed laser.

To fabricate the hybrid perovskite photodetectors, we pattern two silver electrodes with a 2 mm gap atop the pre-coated hybrid film on glass, as shown in the lower inset of Fig. 3a. To study the effect of the SWCNT network morphology on the photodetector performances we have, once again, varied the density of the initial SWCNT film (identified by their electrical resistance values) prior to precursor deposition and conversion. Figure 3a highlights the photocurrent results obtained at 10 V (the complete data are shown in Supplementary Figure S2). All photodetectors are responsive between 300 nm to 800 nm, which matches the MAPbI_3−x_Cl_x_ absorption and concurs with previous reports on perovskite-based photodetectors^19^,^20^,^24^. Moreover, nothing suggests a contribution from the SWCNTs to the charge generation, indicating that their role is limited to collection and transport of the photo-generated carriers produced within the perovskite crystals. In Fig. 3b, we show the variation of the photocurrent at 500 nm as a function of the resistance of the initial SWCNT mesh for all the fabricated devices. Interestingly, we find that the highest photocurrent (1.1 μA) corresponds to an intermediate SWCNT film resistance of ~2 MΩ. This reveals a clear trade-off between having a deeper penetration of the perovskite precursor within the bundled SWCNT film to improve charge collection and, providing sufficient SWCNTs to extract the photo-generated charges and generate the maximum photocurrent. For our best device, we have measured the spectral responsivity (obtained by dividing the photocurrent *I*_*ph*_ at 10 V by the power of the incident light at each wavelength *P*_*λ*_*; R* = *I*_*ph*_*/P*_*λ*_) and the number of electrons generated per each incident photon, also called the photoconductive gain (PG). Both spectra are displayed in Fig. 3c. As expected, the responsivity and the photoconductive gain spectra follow a similar trend as the absorbance of the perovskite (cf. Fig. 3d). At a power density around 10 μW.cm^−2^, we obtain a maximum responsivity of 0.7 A.W^−1^ and a maximum photoconductive gain of 200%. It is worth noting that these values are amongst the highest responsivities reported for perovskite photodetectors illuminated under similar irradiances^1^,^3^,^24^. Meanwhile, Fig. 4a plots the photocurrent of our best hybrid photodetector as a function of the illumination power at 532 nm (see Supplementary Figure S3). The photocurrent increases rapidly at low powers, but saturates at higher powers. Next, we have also calculated the responsivity as a function of the illumination power. As expected, the responsivity is higher at low powers, reaching almost 14 A.W^−1^ around 1.3 μW.cm^−2^. This is more than two orders of magnitude higher than the 0.04 A.W^−1^ responsivity measured at 5 mW.cm^−2^. The lower responsivity at higher powers is due to high recombination rates related to the availability of more shallow traps known to have very short lifetime compared with deep traps states^3^,^4^. This is consistent with numerous reports on photodetectors that involve both quantum dots and perovskites used as photosensitizers^3^,^4^,^25^,^26^. On the other hand, the enhancement of the responsivity at low powers was previously reported on various kinds of photoconductive devices^19^,^27^,^28^. This phenomenon is generally attributed to the limited PG, which is the ratio between the photo-generated carrier lifetime (*τ*_*L*_) to its transit time (*τ*_*T*_). The photo-generated carrier lifetime can be directly determined from the photocurrent decay. Meanwhile, the transit time is defined as the time that a photo-generated carrier takes to travel from one electrode to the other and it is given by 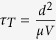, where d, μ and V are respectively the distance between the electrodes, the charge mobility and the applied bias. It is clear that high PG photodetectors need to combine long lifetimes and short transit times. In photodetectors with a distance between the electrodes in the millimeter range, the responsivity is expected to be very low due to the long transit times. Here, the carrier mobility in the bundles of SWCNTs is on the order of 10^5^ cm^2^.V^−1^.s^−1 ^29, which yields very decent photodetector responsivities. One could obviously expect an improvement of the responsivity of our hybrid photodetectors by simply using a much smaller distance between the electrodes, as it was previously demonstrated with hybrid photodetectors based on perovskite and nanocrystalline graphite^1^. To assess the possibility of improving the performances of our photodetectors, we chose to increase the applied bias to reduce the transit time instead of decreasing the distance between the electrodes. Figure 4b shows the typical PL of the hybrid devices as the applied bias ranges from 0 to 73 V. Clearly, the continuous decrease of the PL intensity (see inset of Fig. 4b) with increasing bias confirms that responsivities can be further improved by applying higher voltages and/or reducing the distance between the electrodes.

The stability of our hybrid photodetector was tested by keeping the devices in ambient air, and then the photocurrent spectra were measured at 0, 5, 10 and 20 days. Supplementary Figure S4 shows the typical variation of the photocurrent spectra of the photodetector with aging. After 5 days, the relative decrease of the photocurrent is over 40%, while after 20 days no photocurrent is generated from the device. It is also worth noting that the color of the hybrid perovskite film continues turning to yellow as the device is aging. This indicates that an encapsulation step is required to achieve a long-lasting device.

Finally, by using a nanosecond pulsed laser as photo-generation source, we compared the photocurrent decays of our optimized hybrid device and the device with highest density of SWCNTs. The inset of Fig. 4c shows the transient response of the photodetectors under pulsed laser illumination. The time constants extracted from the exponential decays are respectively 10 ms and 14 ms for our best (2 MΩ) hybrid device and for the device with the lowest SWCNT film resistance (60 kΩ). The shorter time constant observed for the best device confirms the importance of the optimizing the SWCNT network density, but also gives an important insight in the underlying mechanisms yielding higher responsivities. One could argue that a longer time constant with the highest density of SWCNT is due to the overall increase of the number of extraction pathways for the photo-generated carriers. This can be attributed to a lower interpenetration the perovskite precursor within the denser SWCNT networks to form the hybrid SWCNT/perovskite architecture. These time constants are much shorter than previous reports on perovskite hybrid photodetectors, including phototransistors^3^,^4^. To further confirm the photocurrent-time dependence and the reproducibility of the response of our photodetector, we also used a continuous laser (532 nm) modulated at different frequencies (see Supplementary Figure S5). The results reveal the long-term reversibility and stability of the device’s transient photoresponse. The rise-time and fall-time (corresponding to the time interval in which the photocurrent rises from 10 to 90% and falls from 90 to 10%, respectively) are around 13 ms (see Supplementary Figure S6), in agreement with the decay time obtained using the nanosecond pulsed laser.

Our simple two-step processing method for fabricating SWCNT/perovskite hybrids opens a new vista for low-cost and high-performance light-harvesting devices. Indeed, increasing the carrier extraction of perovskite with carbon nanotubes can yield broadband and large-area photodetectors with both high responsivities and unprecedented response times. The high responsivity stems from the high mobility of the SWCNT bundles providing a maximum photoconductive gain of almost 3.10^3^, corresponding to an optimal responsivity of up-to 14 A.W^−1^ (see Supplementary Figure S7). The PG spectrum confirms that the perovskite dominates both the optical absorption and photo-generation, while the SWNT network greatly facilitates carrier separation, transport and collection. Based on these results, our photodetectors can potentially deliver a maximum photoconductive gain of 10^5^, which is two orders of magnitude higher than the highest photoconductive gain reported, even for a bare perovskite phototransistor made with single-crystalline perovskite^19^. Moreover, the response time is more than 1000 times faster than that of hybrid perovskite phototransistors reported so far^4^. This indicates the significant advantages of using carbon nanotubes to enhance the performance of perovskite-based devices and the potential to transform perovskite-based light-harvesting devices.

## Methods

### Materials

All the solutions of SWCNT are COOH-functionalized SWCNT (purity >95% purchased from Nanolab) dispersed in deionized water at a concentration of 0.1 mg.ml^−1^ by sonication for one hour. It is worth mentioning that because of the functionalization, the SWCNTs are mostly p-type. The perovskite precursors, CH_3_NH_3_I (MAI) and PbCl_2_, were purchased form Ossilla. To prepare the MAPbI_3−x_Cl_x_ perovskite, we have used the two precursors MAI and PbCl_2_ at a ratio 3:1. Basically, we have followed the same recipe reported by Binek *et al*.^20^, except that while stirring at 100 °C the solution of 1.685 g of MAI and 973 mg of PbCl_2_ in 4 mL of dry N,N-dimethylformamide (DMF, 99.8%, Sigma–Aldrich), we have added 40 μL of HCl (10% of the volume) to accelerate the dissolution.

Then, after stirring for 30 min, the fully-dissolved precursor solution is spin-coated at 1500 rpm for 30 s directly on the glass substrates pre-coated with SWCNT. Note that perovskite penetrates into the SWCNT network to form a hybrid structure of SWCNT/Perovskite. Next, the glass substrates are heated at 120 °C in air for 15 min to convert the precursors to MAPbI_3−x_Cl_x_ perovskite. It is worth mentioning that before heating the samples, we kept them in air at room temperature until the color of the films start turning to light brown. All the preparation of the solution and the film deposition are performed in ambient air with the average relative humidity level above 50%.

### Material characterization

X-ray diffraction was performed at 1° grazing angle using a Bruker D8 Advanced diffractiometer Cu Kα radiation. The SEM images were obtained using a Hitachi SU 8230 ultra-high resolution field emission scanning electron microscope. For the PL measurements, we used a Torus 532 nm laser (Laser Quantum, Cheshire, UK) for excitation and a Jobin-Yvon iHR320 triple-grating spectrometer (Horiba Scientific, Kyoto, Japan) equipped with a symphony thermoelectrically-cooled Synapse CCD detector array to collect the emission.

### Photocurrent spectrum measurements

The setup used to measure the photocurrent spectrum consists of a Xenon lamp coupled to a TRIAX320 monochromator, a chopper and a lock-in amplifier (see Supplementary Figure S8 for a schematic of the experimental setup). Before exciting the sample, the light from the Xenon lamp passes through the monochromator to perform a 5 nm-step scan from 300 nm to 900 nm. Then, the excitation light is also modulated at 6 Hz prior to illuminate the sample, which is biased at 10 V and placed right after a circular diaphragm of 0.2 cm diameter. Finally, the photocurrent is measured by means of a lock-in amplifier. To calculate the responsivity, we divided the photocurrent by the power of the incident light at each wavelength, which was measured with a calibrated photodiode (Newport 918D) placed at the position of the sample with the same diaphragm aperture. The current-voltage (I-V) characteristics used to extract the photocurrent as a function of the laser power are measured with a Keithley-2400 source-measure unit (SMU). We used a diaphragm with circular aperture of 0.5 cm diameter to define the active area, which is estimated to have a rectangular shape of area 0.2 × 0.5 cm^2^. The transient response was measured using an oscilloscope (Agilent DSO-X 3034A) triggered by the 4 ns pulsed laser (Nd:YAG Quantel Brillant), which is used to illuminate the photodetector. All the photocurrent measurements shown here are representative of 2 to 4 devices, for each given resistance value of the SWCNT film. The photocurrent generated by the different samples is quite reproducible. We found an experimental error between 4% to 6%.

## Additional Information

**How to cite this article**: Ka, I. *et al*. High-performance nanotube-enhanced perovskite photodetectors. *Sci. Rep.* **7**, 45543; doi: 10.1038/srep45543 (2017).

**Publisher's note:** Springer Nature remains neutral with regard to jurisdictional claims in published maps and institutional affiliations.

## Supplementary Material

Supplementary Information
