# Supplementary material for: High-performance nanotube-enhanced perovskite photodetectors
Source: Sci Rep. 2017 Mar 30;7:45543. doi: 10.1038/srep45543 (PMC5371979; doi:10.1038/srep45543)
Supplement: Supplementary Information [file srep45543-s1.pdf]

Supplementary

High-performance nanotube-enhanced perovskite photodetectors

Ibrahima Ka[1]*, Luis Felipe Gerlein[1], Riad Nechache[1]*, Sylvain G. Cloutier[1]*

[1]Department of Electrical Engineering, École de Technologie Supérieure, 1100 Notre-Dame Ouest, Montréal, Québec H3C 1K3, Canada

*Correspondence and requests for materials should be addressed to S.G.C. (email: sylvaing.cloutier@etsmtl.ca), to I.K. (email: ibrahima.ka.1@ens.etsmtl.ca) or to R.N. (email: riad.nechache@etsmtl.ca).

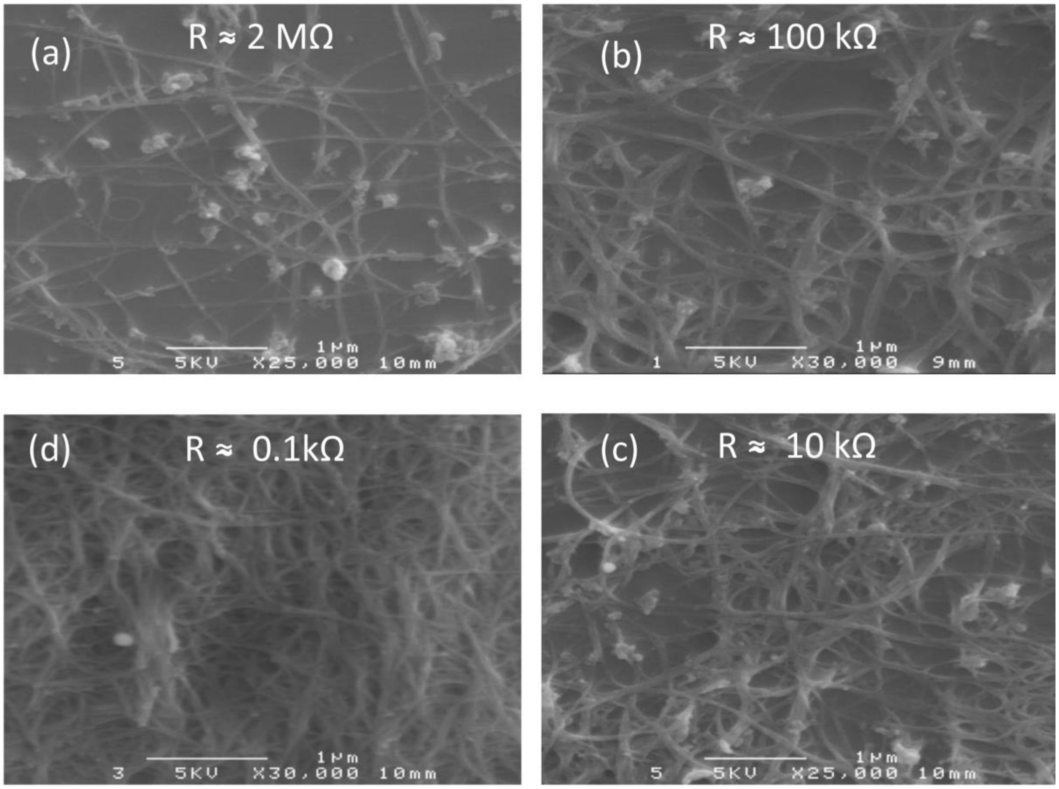

Supplementary Fig. S1. SEM images of SWCNT films with density increasing from (**a**) to (**d**) deposited by spray-coating with their electrical resistance values.

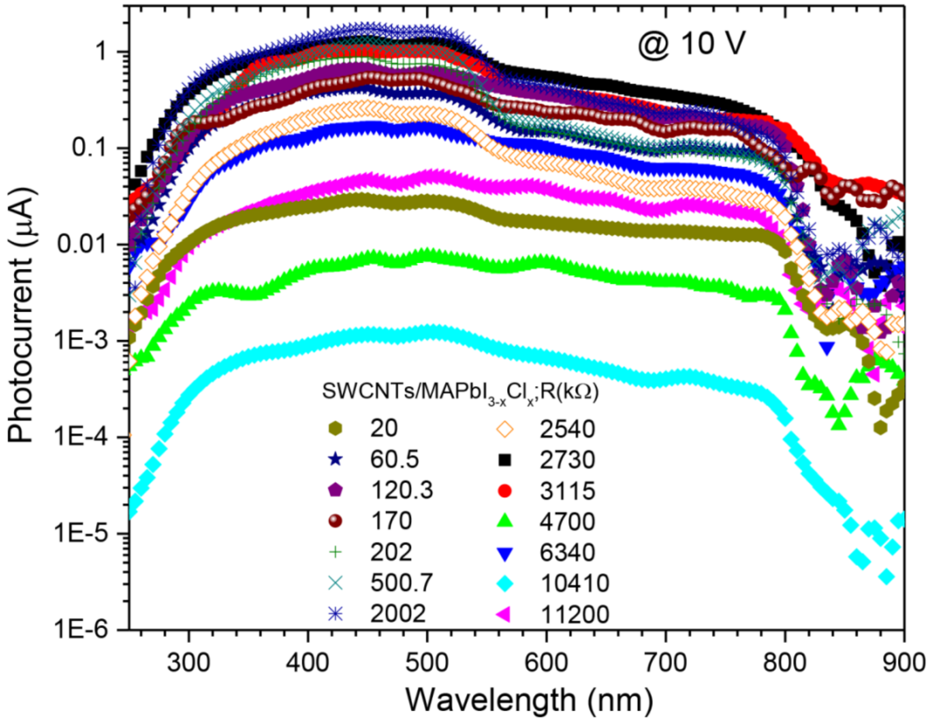

Supplementary Fig. S2. Photocurrent spectrum of all the devices. Photocurrent spectrum of all the devices made with different SWCNT films (identified by their electrical resistance values).

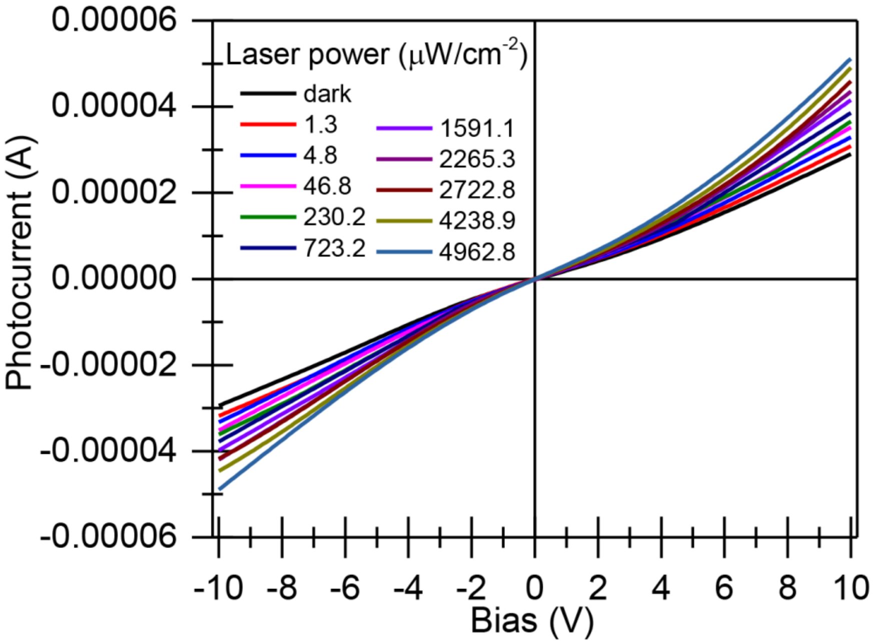

Supplementary Fig. S3. I-V measurements. I-V measurements in dark and under different power densities (varied from 1.3 μW.cm^{-2} to 4.9 mW.cm^{-2}) of the laser at 532 nm. The photocurrent (I_{ph}) at 10 V is calculated using $I_{ph} = I_{under\ laser} - I_{dark}$

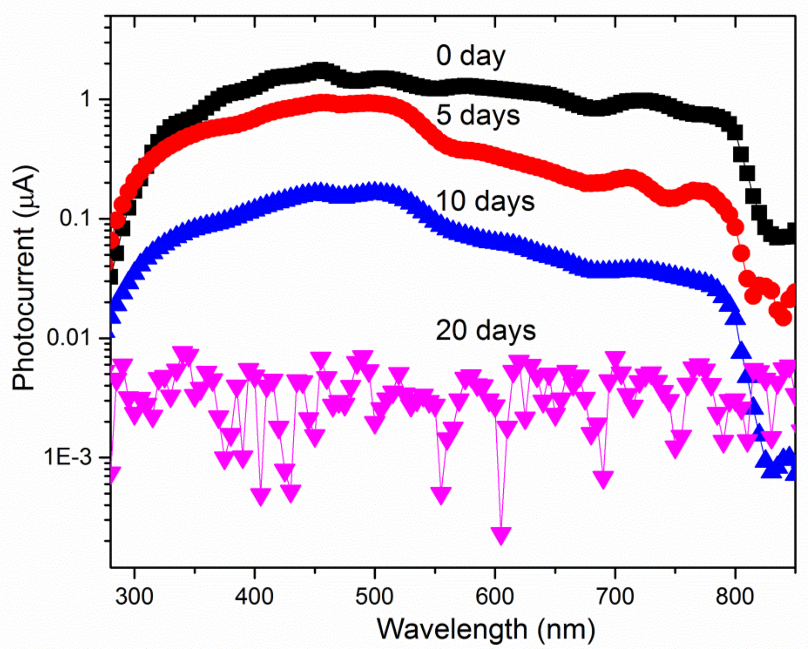

Supplementary Fig. S4. Stability test. Typical variation of the photocurrent spectrum of our device at different ages, showing a constant decrease of the performance as the device is kept in ambient air. These results are representative of the stability of over 80% of the devices.

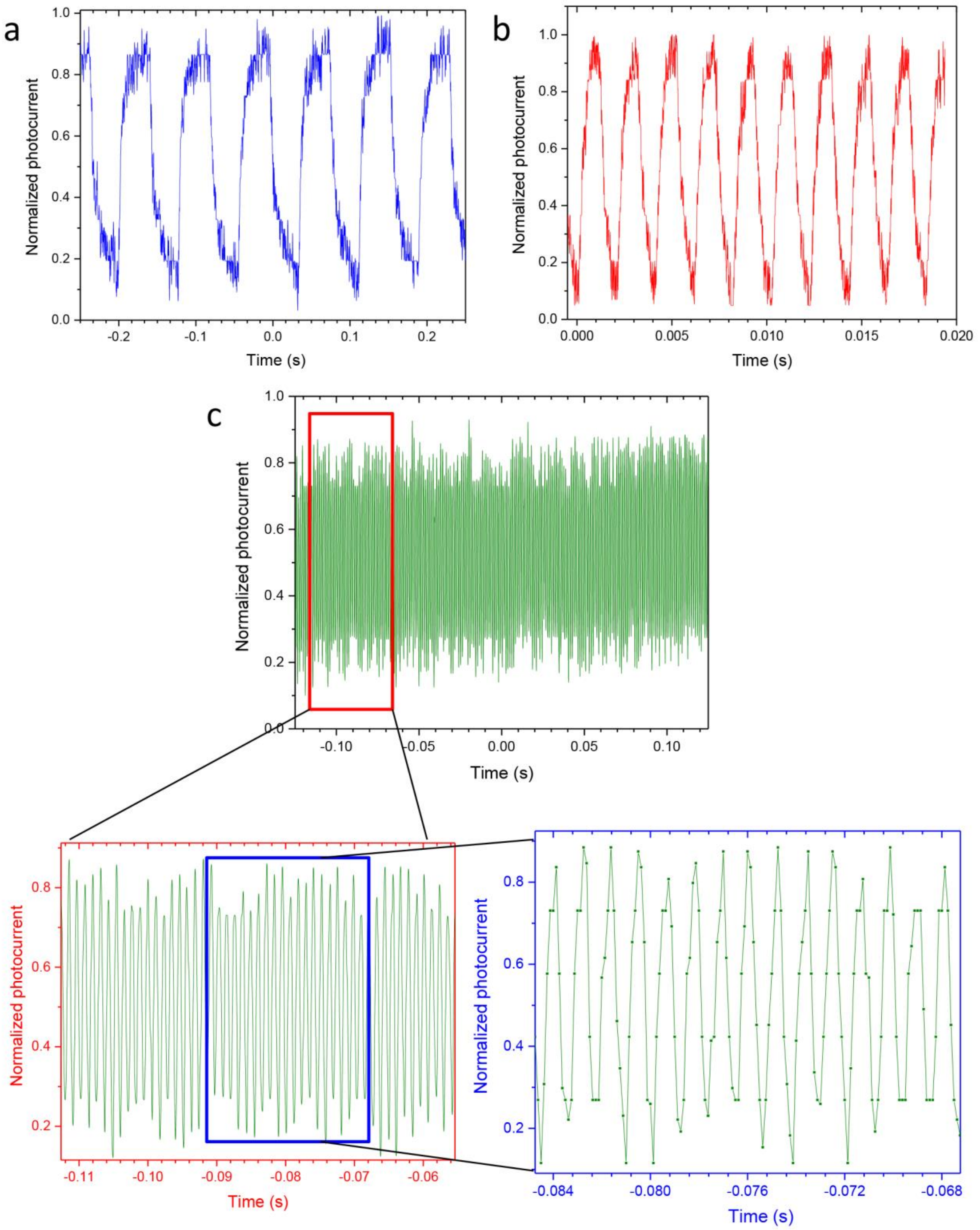

Supplementary Fig. S5. Time-dependent photocurrent. Typical time response measurements of the devices made at 10 V and under repetitive on/off cycles, using a chopper at different frequencies (**a**) 12.5 Hz; (**b**) 620 Hz and (**c**) 1150 Hz. The illumination was a continuous laser at 532 nm.

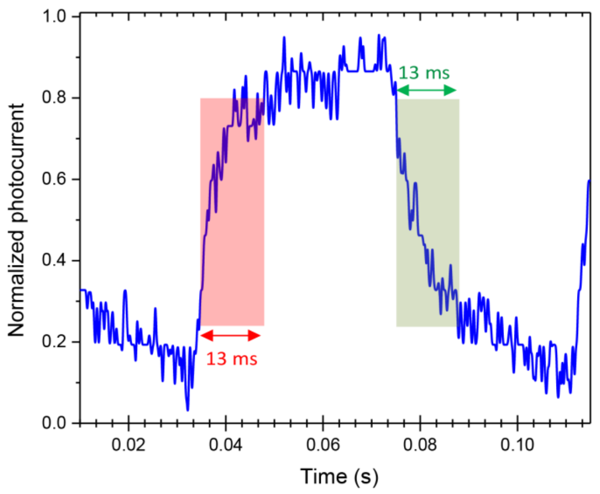

Supplementary Fig. S5. Time response. Rise time and fall time of our photodetector are estimated to be 13 ms.

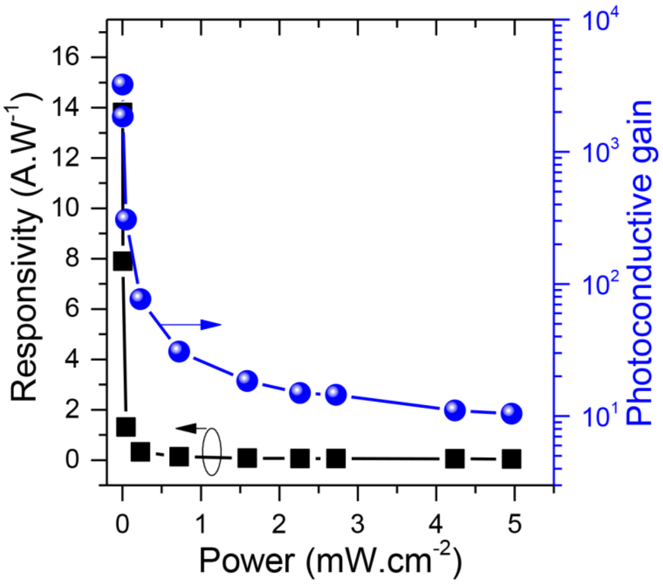

Supplementary Fig. S7. Responsivity and photoconductive gain. Responsivity and corresponding photoconductive gain measured with different illumination powers of the laser at 532 nm.

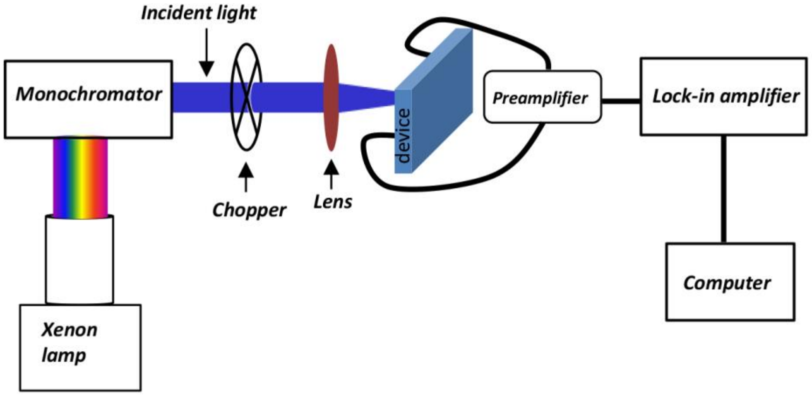

Supplementary Fig. S8. Photocurrent spectrum measurements. Schematic of the experimental setup of the photocurrent spectrum measured under a 10 V applied bias of the hybrid device.

The setup used to measure the photocurrent spectrum consists of a Xenon lamp coupled to a TRIAX320 monochromator, a chopper and a lockin amplifier (see Supplementary Fig. S7 for a schematic of the experimental setup). Before exciting the sample, the light from the Xenon lamp passes through the monochromator to perform a 5 nm-step scan from 300 nm to 900 nm. Then, the excitation light is also modulated at 6 Hz prior to illuminate the sample, which is biased at 10 V and placed right after a circular diaphragm of 0.2 cm diameter. Finally, the photocurrent is measured by means of a lock-in amplifier. To calculate the responsivity, we divided the photocurrent by the power of the incident light at each wavelength, which was measured with a calibrated photodiode (Newport 918D) placed at the position of the sample with the same diaphragm aperture.
